# Efficacy and Safety of Artemisinin-Piperaquine for the Treatment of Uncomplicated Malaria: A Systematic Review

**DOI:** 10.3389/fphar.2020.562363

**Published:** 2020-09-11

**Authors:** Qi Wang, Yuanyuan Zou, Ziyi Pan, Hongying Zhang, Changsheng Deng, Yueming Yuan, Jiawen Guo, Yexiao Tang, Nadia Julie, Wanting Wu, Guoming Li, Mingqiang Li, Ruixiang Tan, Xinan Huang, Wenfeng Guo, Changqing Li, Qin Xu, Jianping Song

**Affiliations:** ^1^ Artemisinin Research Center, Guangzhou University of Chinese Medicine, Guangzhou, China; ^2^ Institute of Science and Technology, Guangzhou University of Chinese Medicine, Guangzhou, China

**Keywords:** artemisinin-piperaquine, meta-analysis, *Plasmodium falciparum*, efficacy, safety

## Abstract

**Objective:**

The World Health Organization recommends artemisinin-based combination therapies (ACTs) for the treatment of uncomplicated malaria to improve the therapeutic efficacy and limit the choice of drug-resistant parasites. This systematic review and meta-analysis aimed to evaluate the comparative efficacy and safety of artemisinin-piperaquine (AP) in the treatment of uncomplicated malaria relative to other commonly used ACTs.

**Methods:**

As per the PRISMA guidelines, the EMBASE, MEDLINE, the Google Scholar Library, and Cochrane library databases were systematically searched from inception until July 2020 with the following terms: “artemisinin-piperaquine” or “AP.” Only randomized controlled trials (RCTs) were included. The competing interventions included dihydroartemisinin–piperaquine (DHA-PPQ), artemether–lumefantrine (AL, Coartem), artesunate-melfloquine (ASAM) and artesunate-amodiaquine (ASAQ, Artekin). Single-arm clinical trial on AP was also assessed. The reported outcomes, including the overall response, cure rate, fever and parasite clearance time, hematology, biochemistry, electrocardiogram (ECG), adverse events, recurrence rate, and sensitivity analyses, were systematically investigated. All data were analyzed using the Review Manager 5.3.

**Results:**

A total of seven studies were reviewed, including five RCTs and two single-arm studies. A pooled analysis of 5 RCTs (n = 772) revealed a comparable efficacy on polymerase chain reaction (PCR)-confirmed cure rate between AP and competing interventions in treating uncomplicated malaria. As for the fever and parasite clearance time, due to the lack of complete data in some studies, only 3 studies’ data could be used. The patients showed good tolerance to all drugs, and some side-effects (such as headache, anoxia, vomiting, nausea, and dizziness) were reported for every group, but they were self-limited and showed no significant difference.

**Conclusions:**

AP appeared to show similar efficacy and safety, with a simpler mode of administration and easier compliance when compared with other ACTs used in the treatment of uncomplicated malaria. Considering that the potential evolution of drug resistance is of a great concern, additional RCTs with high-quality and more rigorous design are warranted to substantiate the efficacy and safety in different populations and epidemiological regions.

## Introduction

Despite malaria being a preventable and treatable infectious disease, as per the World Health Organization (WHO) estimate, approximately half a million people die each year from malaria (WHO, 2019). In fact, malaria mainly affects the lower-and middle-income countries(LMICs)(Godman et al., 2020). Early diagnosis and timely and effective treatment remain the key strategy for reducing malaria-related mortality and morbidity (WHO, 1993). The issue of malaria parasite becoming increasingly resistant to the current medicines has become a global health concern. The data on resistance to routinely used antimalarial drugs, such as sulfadoxine-pyrimethamine, mefloquine, and chloroquine has been reported (Nosten et al., 1991; Ter Kuile et al., 1992; Marfurt et al., 2007; Bruxvoort, 2014; Packard, 2014). WHO recommends ACTs for the treatment of uncomplicated malaria to improve the therapeutic efficacy as well as to limit the drug-resistance of the parasite (Guerin et al., 2002; White, 2002). The rationale for the use of ACTs is based on the fact that artemisinin-based ingredients can rapidly reduce parasitemia, while high concentration of the partner drug can clear the residual parasitemia (Stepniewska et al., 2009).

In a past study, fixed-dose compound tablets AP were used, with each tablet containing 62.5 mg artemisinin and 375 mg piperaquine; this tablet showed good safety and efficacy over a 2-day course of treatment (Trung et al., 2009). Piperaquine is a 4-chloroquinoline compound (1,3-bis[1-(7-chloro-4’-quinolyl)-4’-piperazinyl]) that was used in China in 1970s–80s. Reportedly, as compared with piperaquine phosphate, patients showed better tolerance to piperaquine; moreover, piperaquine could help reduce the treatment expense and time (Davis et al., 2005; Krudsood et al., 2007). A study on the pharmacodynamics of artemisinin and its derivatives yielded that they have the beneficial properties of wide distribution, fast excretion, and absorption *in vivo* (Woodrow et al., 2005).

In endemic countries, data from clinical trials is of great significance as the pattern of malarial transmission varies even over a small distance. Therefore, it is considered worthy to examine the existing reliable evidence to compare the efficacy and safety of AP and other antimalarial drug regimens in endemic countries *via* systematic review of all relevant trials.

## Methods

For the stated study purpose, we followed the standard methods of PRISMA guidelines, which defined the search strategy and the methods employed for data inclusion (Moher et al., 2010).

### Study Search

To evaluate the efficacy of AP in treating uncomplicated malaria, we searched published trials in electronic databases such as the EMBASE, MEDLINE, the Google Scholar Library, the Cairn Library,and Cochrane library. Next, we checked the WHO library database for raw and unpublished data. The reference portions of the selected researches and relevant literature reviews were also examined to discover potential papers. The search was restricted to human studies, published in Chinese, French, and English languages until July 2020. The Medical Subject Headings (MeSH) terms were simply “AP” and “malaria”. Researches were included only when they were randomized controlled trials (RCTs).

### Study Selection

All included trials followed the PICOS standards (Moher et al., 2010).

P: Participants. All participants showed the clinical signs of malaria, such as fever (temperature ≥37.5℃), were of age 7–65 years, and were not taking antimalaria drugs during the previous week. Microscopy of the peripheral blood smear samples detected a parasite count of 200–2,000/µl, indicative of malaria.

I: Intervention. Studies using fixed-dose compound tablets AP were included. The tablets were provided by Artepharm Co., Ltd., China, each tablet contained 62.5 mg of artemisinin and 375 mg of piperaquine.

C: Control. The patients in the control group were taking other fixed-dose antimalarial drugs such artemether–lumefantrine (AL; Coartem), artesunate-amodiaquine (ASAQ), artesunate-mefloquine (ASAM), and dihydroartemisinin–piperaquine (DHP; Artekin).

O: Outcome. The primary endpoints of the trial was efficacy, expressed as PCR-adjusted cure rate. The secondary outcomes were measured based on the parasite clearance time (PCT) and fever clearance time (FCT) and the occurrence of adverse events (AEs). Studies were accepted only if they provided some effect estimates such as the hazards ratio (HR), relative risk (RR), or odds ratio (OR), with 95% confidence interval (CI) for digital computation.

S: Study. Two single-arm studies on AP were also included, which hadn’t been absorbed in the forest figure. After browsing the abstract and full text of this study, we considered 5 articles, all on RCTs.

### Data Extraction and Quality Assessment

Two authors independently reviewed the titles and abstracts yielded from the electronic search. Two reviewers individually collected information (characteristics of each studies, route of administration, and brand of AP) from each included study using the piloted data extraction form.

The Cochrane Collaboration’s risk of bias tool was implemented to define the methodological quality in this study (The Cochrane Colleaboration, 2011; Higgins et al., 2019). The 6 domains for the risk of bias included: free of suggestion of selective outcome reporting, blinding of outcome assessment, allocation concealment, incomplete outcome data adequately addressed, random sequence generation, and other sources of potential bias addressed. Discrepancies between the authors were resolved by adequate discussion. All analyses were conducted by using the intention-to-treat (ITT) method.

### Statistical Analysis

Data were analyzed by estimating the proportions of participants who achieved treatment successfully, which was compared by using the factors summary relative risk (RR), with 95% CI for dichotomous data. RR and 95% CI <1 favored AP for other comparatively antimalaria drug. Heterogeneity in the study outcomes was presented by the I^2^ test.

The value of I^2^ >50% was considered to indicate substantial heterogeneity.

Due to the limited number of data available, no stratification and sensitivity analysis could be performed. All analyses were performed with the RevMan 5·3 (The Cochrane Collaboration, 2014). The protocol followed for this review and meta-analysis has been registered with PROSPERO. Half of the studies did not report standard deviation for the continuous data such as FCT and PCT and hence the data could not be compared.

## Results

### Search Results

**Figure 1:**
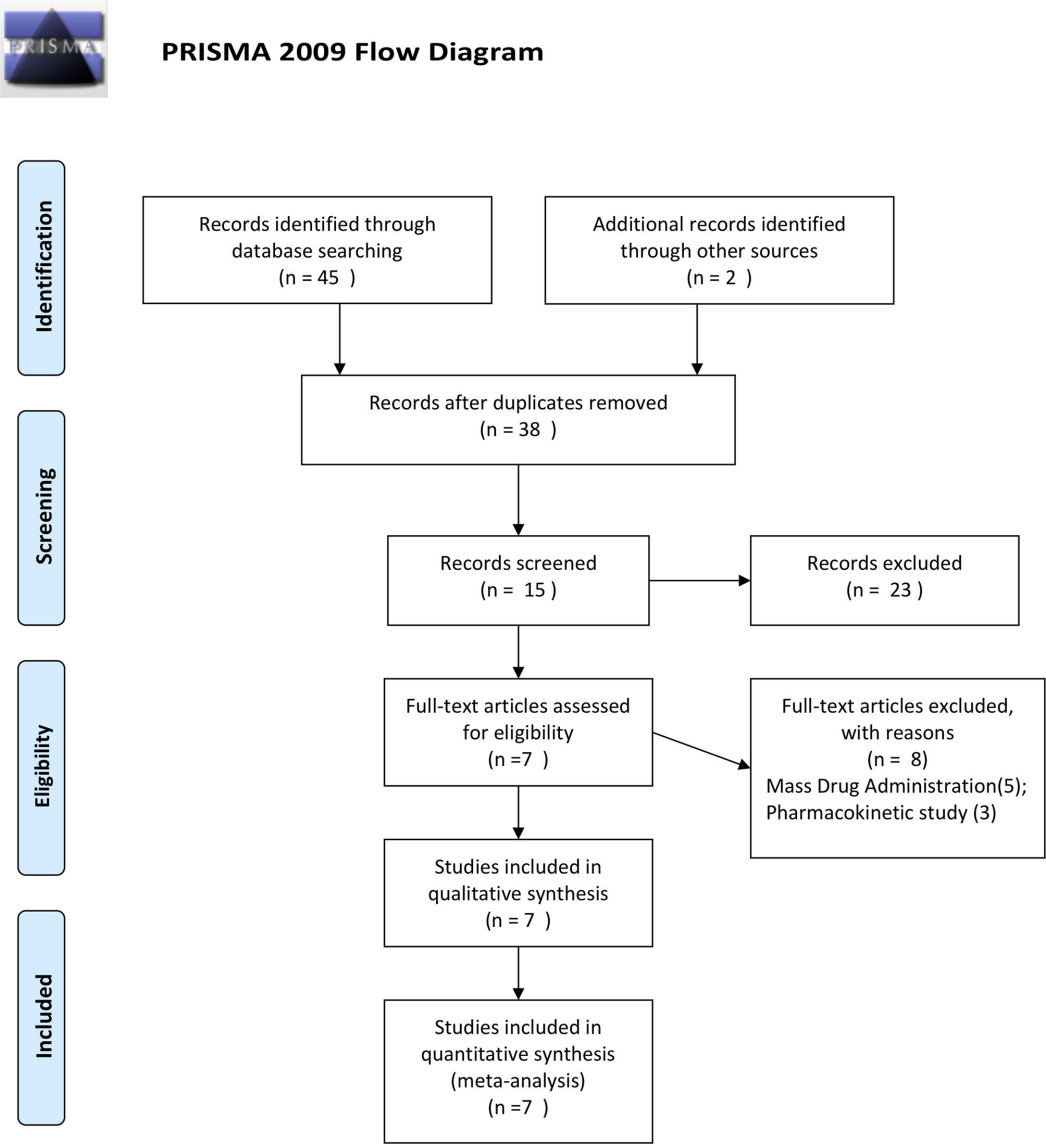
PRISMA flow for data selection.

Our initial search found 45 relevant articles published between 2000 and 2020, of which 9 reported repetitive data. After screening for titles, 38 abstracts, and 15 full-text reviewed, 5 studies reporting RCTs (Krudsood et al., 2007; Trung et al., 2009; Song et al., 2011; Thanh et al., 2012; Onasanya et al., 2015), and 2 reporting a noncomparative single-arm studies (Deng et al., 2008; Wang et al., 2020) were included for the analysis (**Figure 1**).

### Characteristics of the Included Studies

Among the included studies, one was published in the Chinese language (Deng et al., 2008) and others in the English language (Krudsood et al., 2007; Trung et al., 2009; Song et al., 2011; Thanh et al., 2012; Onasanya et al., 2015; Wang et al., 2020). Four of the five RCTs (Krudsood et al., 2007; Trung et al., 2009; Song et al., 2011; Thanh et al., 2012) and the two single-arm studies (Deng et al., 2008; Wang et al., 2020) reported PCR-confirmed cure rate on day 28, while one trial reported the same on day 42 (Onasanya et al., 2015). A total of 917 participants were included in the experiment, including 145 in the single-arm study, 335 in the AP group, 188 in the DHP group, 25 in the ASAM group, 59 in the ASAQ group, and 165 in the AL group. The characteristics of the included single-arm studies are given in **Table 1**.

**Table 1:** Characteristics of the single-arm studies.

Study author, publication year [reference]	Country	Enrolled (n)	Cure rate at day 28	FCT(fever clearance time)	PCT(parasite clearance time)	Recurrence Rate
Deng et al., 2018 Wang et al., 2020	Cambodia Thailand	Adults+children (54) Children (91)	94.4% 98.9%	14.1 ± 7.8 h 32.4 ± 16.2h	60.7 ± 23.9 h 57.3 ± 14,7h	5.55% 1.1%

### Trial Quality

As shown in **Table 2**, half of the studies reported a low risk of bias, which was defined as meeting five of the seven domains (Trung et al., 2009; Thanh et al., 2012). All RCTs included in this review were blinded studies (Trung et al., 2009; Thanh et al., 2012) or open-labeled (Krudsood et al., 2007; Song et al., 2011; Onasanya et al., 2015), which indicates that both the patients and the research investigator may have realized the difference in the treatment of the participants (**Table 2**).

**Table 2:** The risk of bias of the included trials.

Description of domains	Author, publication year				
	Onasanya et al., 2015	Trung et al., 2009	Song et al., 2011	Thanh et al., 2012	Krudsood et al., 2007
Random sequence generation	yes	yes	yes	yes	yes
Allocation concealment	yes	yes	yes	yes	yes
Blinding of outcome assessment	unclear	unclear	unclear	yes	unclear
Blinding of participants and personnel	Open label	yes	Open label	yes	Open label
Incomplete outcome data adequately addressed	yes	yes	yes	yes	yes
Free of selecting outcome reporting*	unclear	yes	yes	yes	yes
Addressed other sources of potential bias	unclear	unclear	unclear	unclear	unclear

Yes, low risk of bias; Unclear, uncertain risk of bias; Open label, high risk of bias.

### Efficacy

**Figure 2:**
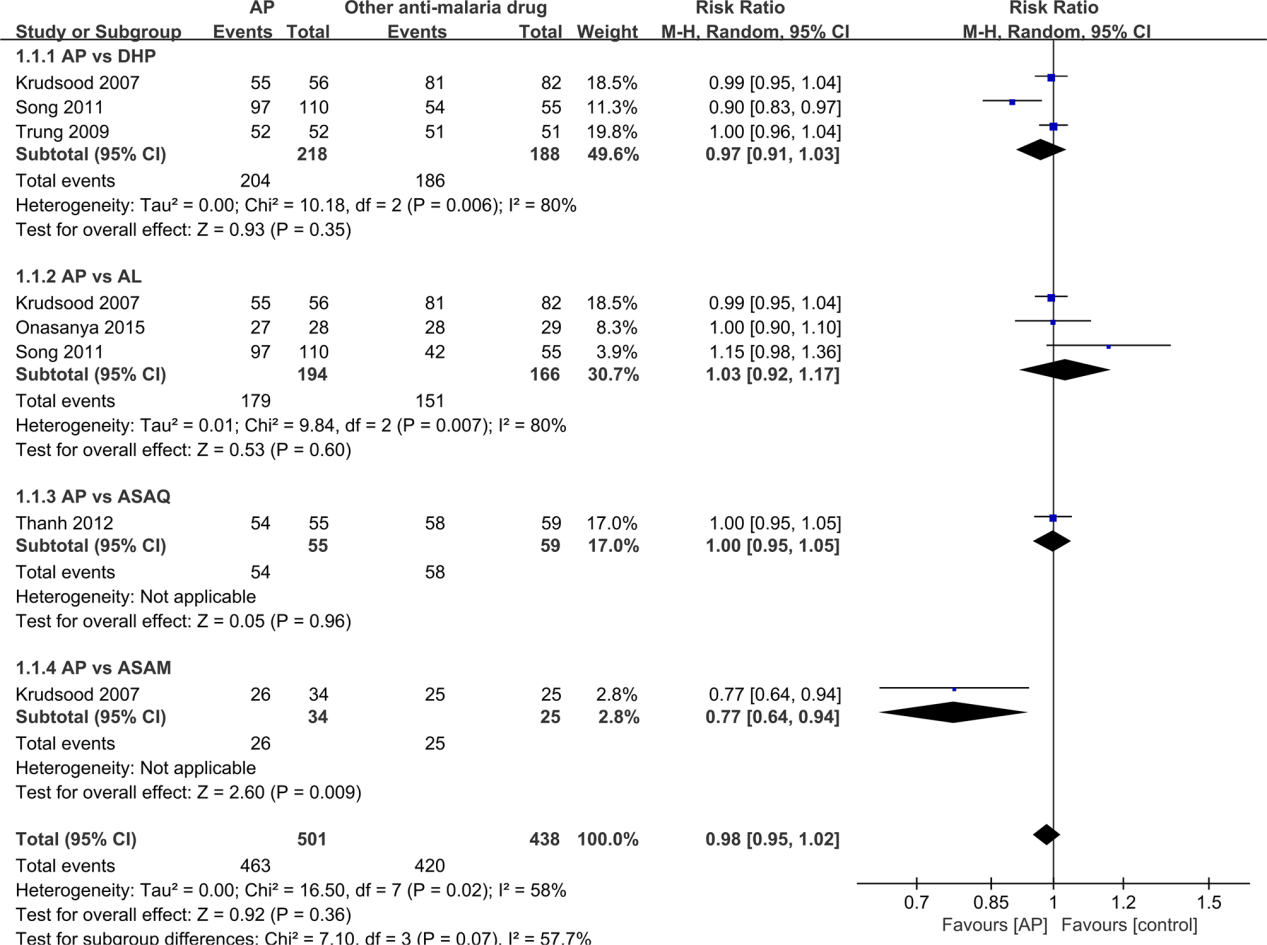
Comparative drug efficacy between artemisinin–piperaquine and competing interventions.

It was unrealistic to conduct a pooled analysis as the RCTs included in the current review did not compare AP with only one antimalarial drug. There was a similar primary efficacy endpoint between AP (98.2%, 54/55) and ASAQ (98.3%, 58/59) based on the PCR results (RR: 1.0, 95% CI 0.95–1.05) (Thanh et al., 2012), between AP (93.6%,204/218) and DHP (98.9%, 186/188) on PCR-adjusted cure rate (RR:0.97, 95% CI 0.91-1.03) (Krudsood et al., 2007; Trung et al., 2009; Song et al., 2011), between AP (92.3%; 179/194) and AL (91.0%, 151/166) on PCR-adjusted cure rate (RR: 1.03, 95% CI 0.92-1.17) (Krudsood et al., 2007; Song et al., 2011; Onasanya et al., 2015), and between AP (76.5%, 26/34) and ASAM (100%, 25/25) on PCR results (RR: 0.77, 95% CI 0.64–0.94) (Krudsood et al., 2007) (**Figure 2**).

### FCT and PCT

**Figure 3:**
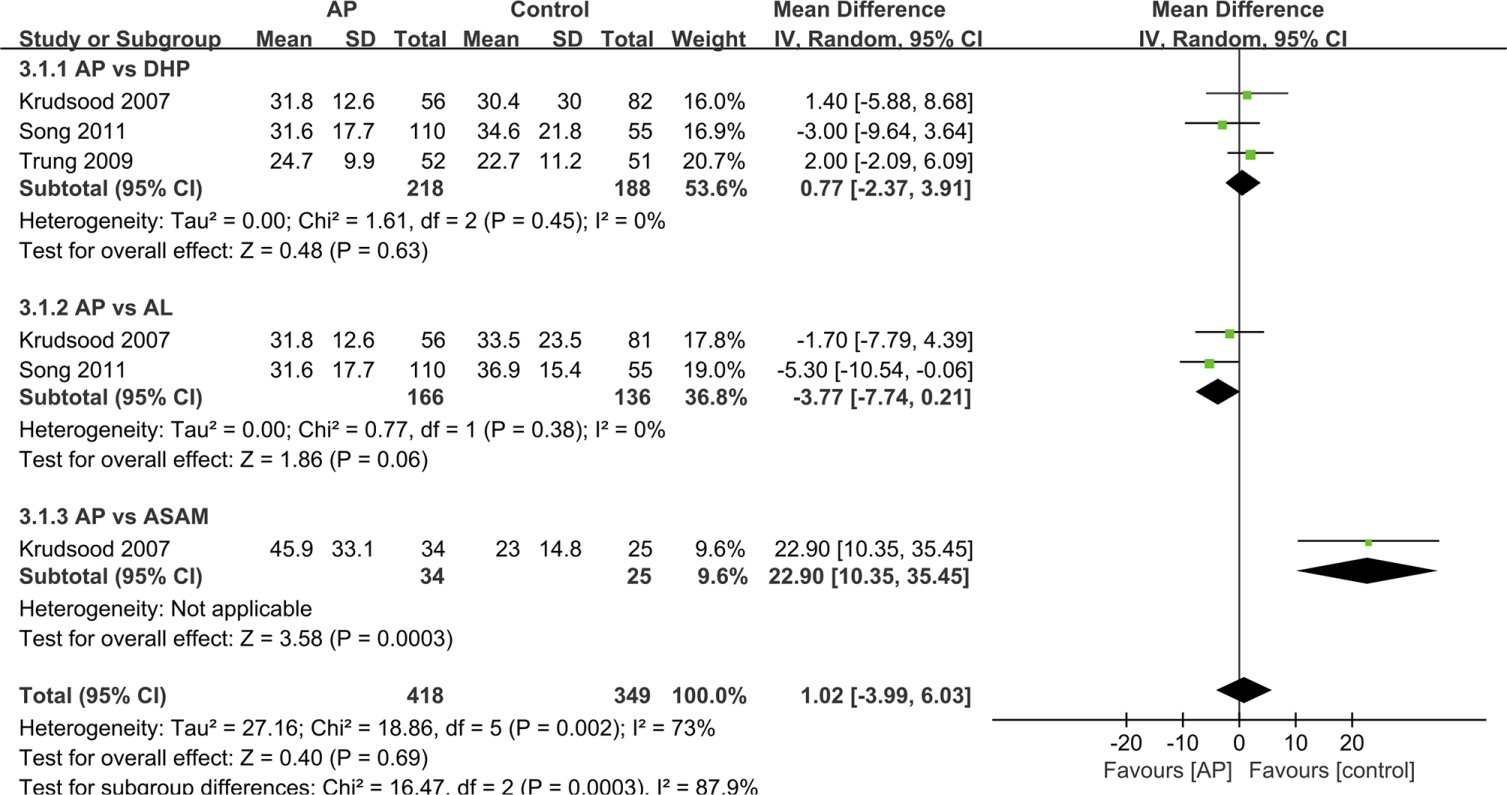
Comparative fever clearance time (FCT) between ATQ and competing interventions.

**Figure 4:**
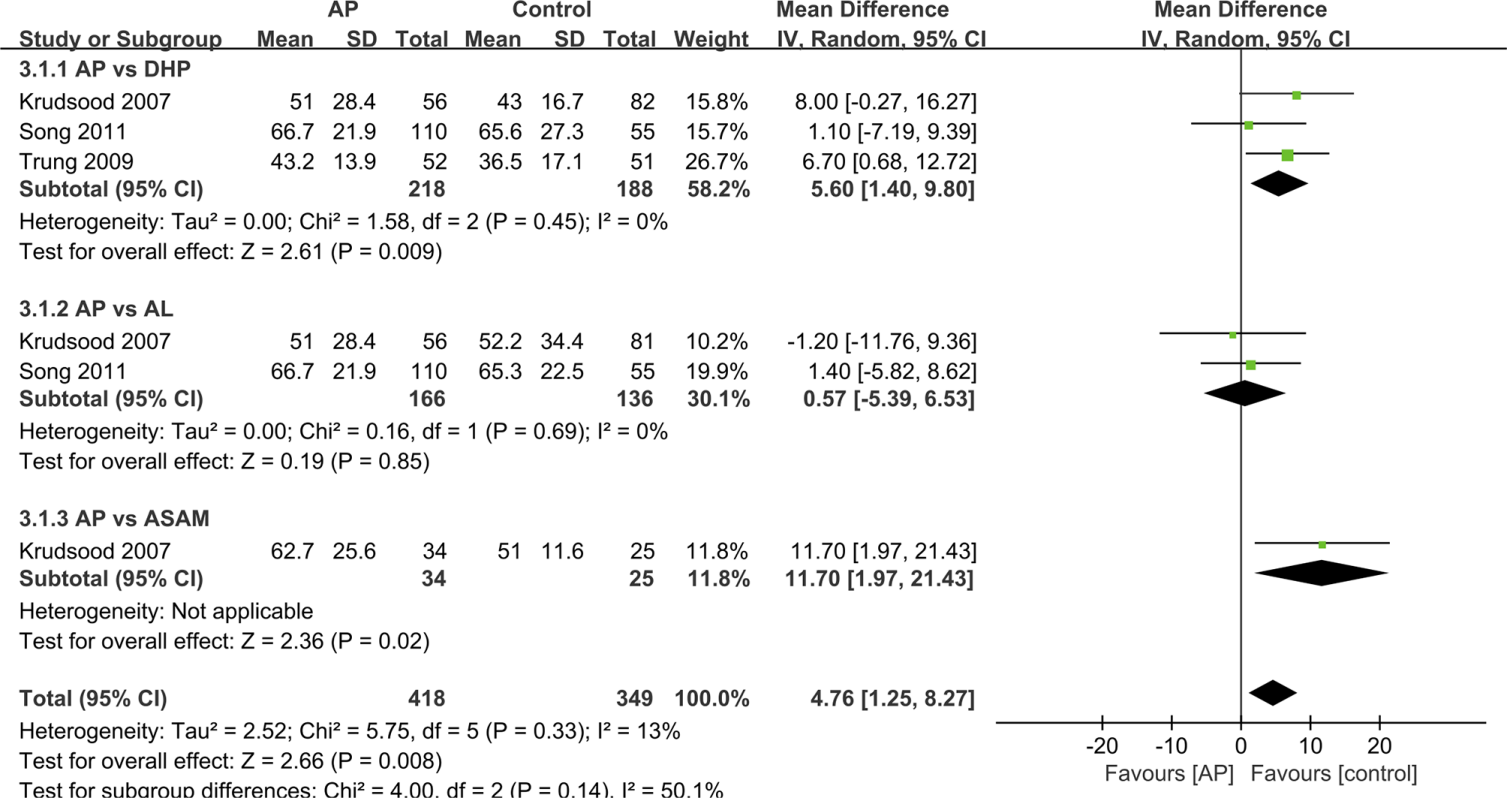
Comparative parasite clearance time (PCT) between ATQ and competing interventions.

We analyzed the data that did not find a suitable form in the form of a table. For other studies, we made forest maps for analysis. The FCT and PCT reported in two trials (Thanh et al., 2012; Onasanya et al., 2015), the difference between the control group and the experimental group was not significant, and the two sets of data were not merged into the forest plot. (**Table 3**) And the FCT and PCT of other studies (Krudsood et al., 2007; Trung et al., 2009; Song et al., 2011) give the mean ± standard deviation, which we present in the forest plots for analysis (**Figures 3** and **4**).

**Table 3:** Parasite and fever clearance time (h) of two trials.

Study author, publication year [reference]	Country	Included population	Drug(n)	Fever clearance time	Parasite clearance time
Onasanya et al., 2015	Nigeria	Adults	ATQ (28)	12 h	48 h
			Coartem (29)	24 h	24 h
Thanh et al., 2012	South-central Vietnam	Adults+children	ATQ (55)	24 h	48 h
			ASAQ (59)	12 h	36 h

ATQ, Artemisinin-piperaquine; Coartem, Artemether-lumefantrine; ASAQ, Artesunate-amodiaquine.

The PCT was significantly longer in the AP group as compared with that in the ASAQ group (48 h vs. 36 h, P < 0.001) (Thanh et al., 2012). The mean PCT in DHP was shorter than that in the AP group (MD=5.60 95%CI 1.40–9.80 Z=2.61 P=0.009<P=0.05) (Krudsood et al., 2007; Trung et al., 2009; Song et al., 2011). The median PCT was remarkably slower in the ASAM group as compared with that in the AP group (MD=11.70 95%CI 1.97–21.43 Z=2.36 P=0.02<P=0.05) (Krudsood et al., 2007). Only one outcome showed that the PCT had no significant difference between AP and AL group(MD=o.57 95%CI −5.39–6.53 Z=0.19 P=0.857>P=0.05) (Krudsood et al., 2007; Song et al., 2011). Overall, the difference in PCT between the AP and control groups is statistically significant (MD=4.76 95% CI 1.25-8.27 Z=2.66 P=0.008<P=0.05).

As for the FCT, the data showed difference between the results for ASAQ and AP groups (12 h vs. 24 h, P = 0.07) (Thanh et al., 2012) or between those of the DHP and AP groups (12 h vs. 24 h, P < 0.05) (Onasanya et al., 2015). And studies presented no significant difference between the AP and the DHP group (MD=0.71 95% CI −2.37–3.91 Z=0.48 P=0.63>P=0.05) (Krudsood et al., 2007; Trung et al., 2009; Song et al., 2011). As for the comparison between AP group and AL group, AP group shows an advantage in FCT (MD=−3.77 95% CI −7.74–0.21 Z-1.86 P=0.06>P=0.05) (Krudsood et al., 2007; Song et al., 2011). However, in the comparison between AP and ASAM groups, AP group shows a disadvantage in FCT (MD=22.9 95% CI 10.35–35.45 Z=3.58 P=0.00003<P=0.05) (Krudsood et al., 2007). In general, the difference in FCT between AP group and other antimalaria group is not obvious (MD=1.02 95% CI −3.99–6.03 Z=0.4 P=0.69>P=0.05).

### Hematology, Biochemistry, and ECG

The records of three RCTs (Krudsood et al., 2007; Trung et al., 2009; Song et al., 2011) were analyzed. Two studies showed no abnormal changes in the biochemical and hematological results before and after the treatments in both the groups (Krudsood et al., 2007; Trung et al., 2009). The other trial showed no significant changes in the hematology and ECG results of the three study groups (Song et al., 2011). Two single-arm studies also showed no hematological or ECG changes in the examination (Deng et al., 2008; Wang et al., 2020). However, some indicators of slightly abnormal ECG results at day 7 were recorded, but they returned to the normal level at day 14 (Deng et al., 2008; Song et al., 2011).

### Adverse Events

In the two RCTs analyzed, AEs were reported and compared among the treatment groups, as shown in **Table 4**. Because of the inconsistent findings between the reports, undertaking synthetic estimations of AE incidence was difficult. The most commonly recorded adverse reactions that were considered to be drug-related included nausea, tiredness, vomiting, and headache (**Table 4**).

**Table 4:** Comparison of adverse events of included trials.

Study author, publication year [reference]	Included population	Competing drugs(n)	Adverse events (ATQ vs. Competing interventions)
Onasanya et al., 2015	Adults	Coartem (29)	NA
Thanh et al., 2012	Adults+children	ASAQ (59)	Headache (1.6% vs. NA) Tiredness (1.6% vs. NA) Anorexia (1.6% vs. NA)
Song et al., 2011	Adults+children	DHP (55)	NA
		AL (55)	NA
Trung et al., 2009	Adults+children	Artekin	Dizziness (9.6% vs. 7.8%, P = 0.976) Nausea (9.8% vs. 5.8%, P = 0.692) Vomiting (0% vs. 9.8%, P = 0.063) Anorexia (0% vs. 2.0%, P = 0.992)
Krudsood et al., 2007	Adults	ASAM(25)	Headache (8.8% vs. 48%) Dizziness (8.8% vs. 28%) Vomiting (5.9% vs. 8%)
		AL(81)	Headache (25% vs. 6.2%) Dizziness (7.1% vs. 6.2%) Vomiting (0% vs. 0%)
		DHP(82)	Headache (25% vs. 9.8%) Dizziness (7.1% vs. 8.5%) Vomiting (0% vs. 4.9%)

ATQ, Artemisinin-piperaquine; Coartem, Artemether-lumefantrine; ASAQ, Artesunate-amodiaquine; DHP, Dihydroartemisinin-piperaquine; AL, Artemether-lumefantrine; Atekin, Dihydroartemisinin–piperaquine; NA, not available.

### Recurrence Rate

**Figure 5:**
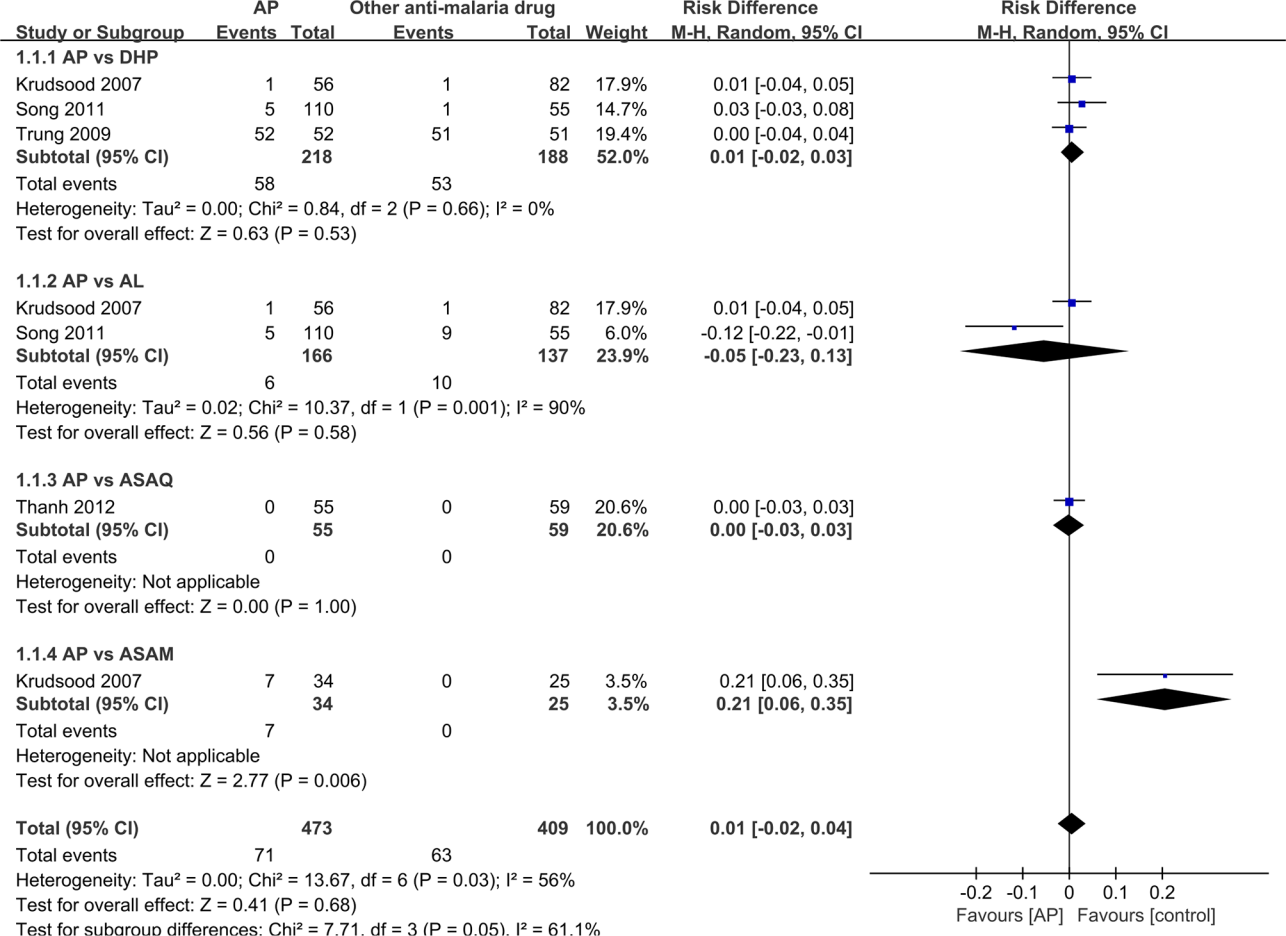
Comparative drug recurrence rate between artemisinin–piperaquine and competing interventions.

Four of the RCTs as well as two single-arm studies exhibited data on recurrence rate, with the re-infected data removed. As two studies included zero event (Krudsood et al., 2007; Thanh et al., 2012), risk difference (RD) was used to analyze the data, as depicted in **Figure 5**. There shows no significant difference between AP and DHP group (RD:0.0, 95%CI −0.02–0.03, P=0.53>P=0.05) (Krudsood et al., 2007; Trung et al., 2009; Song et al., 2011), between AP and AL group (RD:−0.05, 95%CI −0.23–0.13 P=0.58>P=0.05) (Krudsood et al., 2007; Song et al., 2011), and between AP and ASAQ (RD:0.00, 95%CI −0.03–0.03, P=1>P=0.05) (Thanh et al., 2012). However, in one study, the ASAM group shows superiority when compared to AP group ((RD:0.21, 95%CI 0.05–0.35, P=0.006 < P=0.05) (Krudsood et al., 2007). The pooled analysis of 4 RCTs (n = 715) demonstrated no significant difference between the AP and competing groups (RD:0.01, 95%CI −0.02–0.04;Z=0.01, P=0.68>P=0.05) (Krudsood et al., 2007; Trung et al., 2009; Song et al., 2011; Thanh et al., 2012).

### Sensitivity Analyses

For every RCTs analyzed, all patients who were lost to follow-up or had withdrawn from the study were removed from the denominator. As a result, the 95% CI became board (RR, 0.98, 95%CI 0.95 –1.02), and heterogeneity is a bit high among all the studies(I^2^ = 58%).

On account of the limited amount of trials, sensitivity analysis could not be conducted. We did not investigate the publication bias as 10 studies is the minimum recommended number for the same (Higgins et al., 2019).

## Discussion

To further the advancements in designing therapeutic strategies for the development of effective antimalaria administrations, systematic monitoring of the efficacy, safety, and resistance of antimalaria drugs is a must. To the best of our knowledge, this is very first comprehensive analysis of AP for the treatment of uncomplicated malaria. As an individual study is insufficient to yield a valid conclusion, meta-analysis was performed by combining the results of previous studies to compare the efficacy and safety of the new antimalarial AP drug in treating uncomplicated malarial patients. We found similar efficacy and safety of AP relative to other antimalarial drugs available for the treatment of clinical malaria patients living in the endemic regions and countries, including Nigeria, Vietnam, and Cambodia–Thailand border area.

However, AP is a kind of new drug and there are not many RCTs studies about it, most of the randomized controlled clinical trials included in this study were with small sample sizes. Nevertheless, the analysis of the pooled data indicated a comparable efficacy between AP and other antimalarial drugs such as ASAQ, DHP, and AL.

As compared with the cure rate of 14 days, 28 days of parasitological cure rate may act as a more sensitive indicator for the efficacy of antimalarial drugs *in vivo*. Therefore, in the current analysis, the end-point of PCR-corrected cure rate at day 28 was an acceptable outcome marker to increase the sensitivity of the test as well as to generate appropriate data for the comparison of different drug therapy regimens. As a result, all RCTs used the best dose of AP included in the current review and two single-arm studies together demonstrated an efficacy of >95%, which is a desirable standard recommended by the WHO for a new ACT (WHO, 2001). It’s worth noting that there is a research subgroup aimed to determine the optimal therapeutic dose of AP, which was not used during the comparison with the ASAM group (Krudsood et al., 2007).

Although only three studies shared data of standard deviation of FCT and PCT, meta-analysis to assess the risk of AP on the PCT and FCT could still be performed. The overall results from the analysis is that there is no significant difference between AP and AL groups; between AP and DHP groups and between AP and ASAQ groups. Although there is a significant difference between AP and ASAM groups, the AP group does not use the optimal dose, so this data is of little significant to this meta-analysis.

The AL trial in the Cambodia–Thailand border area recorded a slightly high recurrence rate (Song et al., 2011). Reportedly, this may be attributed to the fact that the patient was deficient in fat in his/her diet, which affected the absorption of lumefantrine in AL. In fact, it was proved that dietary fat could improve the bioavailability of AL, although this effect was more pronounced on lumefantrine. As compared with that in the fasting state, administrating AL to healthy volunteers along with a high-fat diet increased the bioavailability of artemether by a factor of 2 and that of lumefantrine by a factor of 16 (White et al., 1999; Ezzet et al., 2000; Djimdé and Lefèvre, 2009).

In Thailand, Krudsood et al. (2007) conducted a dose-ranging study, where he found that a 3-day course of AP was more effective than a 2-day regimen of AP (75% vs. 98% for PCR-adjusted cure rates) for the treatment of uncomplicated malaria patients, with a 28-day follow-up.

### Schedule of Treatment

Combination formula products are often preferred because they reduce the availability and use of monotherapy, and this in turn may reduce the development of resistance(Godman et al., 2020; Neena et al., 2010; Baiden et al., 2015; Mori et al., 2016; Ebenebe et al., 2018). In a multicenter clinical trial in Africa, 92.0% of cases of uncomplicated falciparum malaria were cured (Sagara et al., 2009). In another multicenter trial (Zwang et al., 2009), DP was found to be well-tolerated, effective, easier to administer, and provided better post-treatment prevention than any of the alternative ACTs investigated (Such as mefloquine in Thailand, Myanmar, Laos and Cambodia, AL in Uganda, and amodiaquine-SP and AS-amodiaquine in Rwanda). Among African children from different local environments, a study (Bassat et al., 2009) found that DP was as effective as AL in the treatment of uncomplicated malaria, with similar safety (during a 42-day follow-up, The incidence of recurrent parasitic diseases in group DP is much lower). However, Burkina Faso issued a slightly worrying report. In a study involving 559 children, 31.2% of children treated for AL and 7.6% of children treated for DP. A “new” parasitic disease was developed during the 42-day follow-up (Some´ et al., 2010), although no attempt was made to use genotyping to distinguish between reinfection and true treatment failure. In their Cochrane review (Sinclair et al., 2009), the study showed that 5% of people taking DP in Africa failed treatment. DP is more effective than most other antimalarial ACTs currently widely used. In terms of tolerability and safety, DP seems to have more favorable conditions compared with other ACTs. Sinclair et al. (2009) pointed out that, for example, the incidence of abdominal pain and headache of DP is lower than that of AL, and symptoms such as sleep disturbance, dizziness, anxiety, nausea, and vomiting are all rarer than AS-mefloquine after DP. Although DP is generally used without any specific food-related instructions, data are showing that the combination with fat (such as milk or biscuits) can improve the bioavailability and possible efficacy of piperaquine (Ezzet et al., 2000; Price et al., 2009). During the treatment of ACTs, most adverse reactions are considered to be caused by partner drugs, and AL and DP are well-tolerated drug regimens. The risk of anorexia, nausea, vomiting, and dizziness in ASAQ and ASAM is higher than that of AL or DP (Saito et al., 2020). Artemisinin is currently the only antimalarial drug that has not caused clinical resistance, and it is still effective against multidrug-resistant falciparum malaria. The clinical elimination half-life of these derivatives is several minutes to several hours. As a monotherapy, at least 7 days of artemisinin treatment is required. Piperaquine is a highly fat-soluble drug with the characteristics of large distribution volume, steady state/high bioavailability, and long elimination half-life. The average terminal elimination half-life (T1/2) is very long (543 h) (Davis et al., 2005). These characteristics, together with its tolerability, effectiveness and low cost, make piperaquine an excellent potential cooperative drug for ACTs. There are Mass Drug Administration (MDA) with AP showed that AP can effectively reduce malaria prevalence, and no serious adverse reactions have been reported (Davis et al., 2005; Deng et al., 2018). Research has found that AP+PMQLD (low-dose primaquine) vs. AP alone showed effectiveness of >99% for both therapies, with no substantive contribution of PMQLD on MDA outcomes, which indicate that treat uncomplicated malaria with AP alone is expected to improve compliance (Deng et al., 2018).

### Study Limitations

We acknowledge some limitations in the present study, including that studies published in languages other than French, English and Chinese were missed. Future research needs to address the cost-effectiveness of the antimalarial drug interventions. Moreover, some methodological difficulties were encountered in the present work during the pooling of results. For example, large differences in data reports made it difficult to compare changes in the assessed hemoglobin and ECG levels. In addition, the follow-up period was far too short, because of which a minimum of 42 days was required for forms of ACTs (ANZCTR; WHO, 2010: http://www.ANZCTR.org.au).

All RCTs assessed reported a small sample size and poor methodological quality. Therefore, there is a concern regarding confidence in the treatment effect estimates. Moreover, the risk of performance bias is a concern due to the default blinding in some of the included RCTs (Song et al., 2011; Onasanya et al., 2015).

## Conclusions

This review demonstrates the comparative efficacy of AP with other competing ACTs, such as AL, DHP, ASAM and ASAQ for the treatment of uncomplicated malaria. Based on the review, a better post-treatment prophylactic effect due to the longer elimination half-life of piperaquine (23–33 days), simplicity of administration, and good compliance of AP signifies the potential of becoming a first-line antimalarial drug. However, considering the potential factor of drug resistance, adequately powered, larger and well-designed studies are recommended to confirm the safety and efficacy of AP in different epidemiological settings and different populations.

## Author Contributions

Original idea by QW and JS. YZ carried out the review and meta-analysis with guidance from XH, WG, CL, and QX. ZP, HZ, YY, JG, YT, CD, NJ, WW, GL, ML, and RT drafted the manuscript and the remaining authors contributed with additions and amendments. All authors contributed to the article and approved the submitted version.

## Funding

This work was supported by Natural Science Foundation of China [Grant Number 81873218], Guangzhou Provincial Science and Technology Program [Grant Number 201807010007], China post-doctoral science foundation [Grant Number A2-2902-19-414-007], National “major new drug innovation and development” science and technology project of Ministry of science and technology, People Republic of China [Grant Number 2018ZX09303008], and prevention and control of novel coronavirus infection science and technology project of Guangdong province [Grant Number 2020A111128013].

## Conflict of Interest

The authors declare that the research was conducted in the absence of any commercial or financial relationships that could be construed as a potential conflict of interest.
